# Ultrasound-Guided Hydrodissection for Carpal Tunnel Syndrome with Bifid Median Nerve and Persistent Median Artery: An Imaging-Based Case Report with Alpha-2 Macroglobulin

**DOI:** 10.3390/diagnostics16091362

**Published:** 2026-04-30

**Authors:** Jeimylo C. de Castro, Daniel Wang, Jeffrey Strakowski, Yonghyun Yoon

**Affiliations:** 1 SMARTMD Clinic for Non-Surgical Interventions Pain Center, Centuria Medical Makati, Makati 1205, Philippines; 2 Elohim Research Center, Adventist University of the Philippines, Silang, Cavite 4118, Philippines; 3 Miller School of Medicine, University of Miami, Miami, FL 33136, USA; 4 University of Miami Health System, Miami, FL 33136, USA; 5 Department of Physical Medicine & Rehabilitation, The Ohio State University, Columbus, OH 43214, USA; jstrak@aol.com; 6 Department of Orthopaedic Surgery, Gangnam Sacred Heart Hospital, College of Medicine, Hallym University, 1 Singil-ro, Yeongdeungpo-gu, Seoul 07441, Republic of Korea

**Keywords:** carpal tunnel syndrome, bifid median nerve, persistent median artery, ultrasound-guided hydrodissection, alpha-2 macroglobulin, case report

## Abstract

**Introduction:** Carpal tunnel syndrome (CTS) is the most common entrapment neuropathy of the upper extremity and results from compression of the median nerve within the fibro-osseous carpal tunnel. Anatomical variants such as a bifid median nerve (BMN) and a persistent median artery (PMA) may increase tunnel occupancy and complicate both diagnosis and treatment. High-resolution musculoskeletal ultrasound enables detailed evaluation of these anatomical variations and facilitates image-guided interventions. Ultrasound-guided hydrodissection has emerged as a minimally invasive technique capable of mechanically releasing perineural adhesions and restoring nerve mobility. Alpha-2 macroglobulin (A2M), an autologous plasma protease inhibitor with anti-inflammatory and cytokine-binding properties, has recently been explored as a biologic adjunct in musculoskeletal conditions. **Case Presentation:** We report the case of a 60-year-old right-handed woman who presented with a one-year history of numbness, paresthesia, and pain within the median nerve distribution of her dominant hand. Ultrasound examination demonstrated a bifid median nerve accompanied by a persistent median artery and perineural edema within the proximal carpal tunnel. The patient underwent three weekly sessions of ultrasound-guided hydrodissection using autologous A2M prepared through the APEX filtration system. The patient reported progressive clinical improvement following treatment. Grip strength increased from 12 kg at baseline to 22 kg at week twelve. Follow-up ultrasound performed ten months after treatment showed restoration of median nerve fascicular architecture and normalization of nerve morphology, findings consistent with interval structural improvement. **Discussion:** This case highlights the role of ultrasound in the integrated evaluation and management of CTS with anatomical variants, including diagnosis, procedural guidance, and longitudinal assessment. **Conclusions:** Ultrasound-guided hydrodissection with A2M may represent a feasible minimally invasive approach in selected patients; however, further prospective studies are required to determine its safety and therapeutic efficacy.

## 1. Introduction

Carpal tunnel syndrome (CTS) is the most prevalent entrapment neuropathy affecting the upper extremity and has been reported to affect approximately 3–6% of the adult population worldwide. The condition arises from compression of the median nerve within the carpal tunnel, a confined fibro-osseous space bounded dorsally by the carpal bones and volarly by the transverse carpal ligament. Increased intracarpal pressure may lead to ischemia of the median nerve, impaired axonal transport, and inflammatory changes, resulting in sensory disturbances and motor dysfunction of the hand [1].

The median nerve originates from the medial and lateral cords of the brachial plexus and receives contributions from nerve roots C5 through T1. As it traverses the carpal tunnel beneath the transverse carpal ligament, the nerve normally appears as a single trunk before dividing distally into terminal sensory and motor branches that innervate the thumb, index finger, middle finger, and radial half of the ring finger as well as the thenar musculature.

Although repetitive hand activity, systemic metabolic disorders, and inflammatory conditions are well-recognized contributors to CTS, anatomical variations in the median nerve and surrounding vascular structures may also influence the pathophysiology of CTS. One such variation is the bifid median nerve (BMN), characterized by division of the median nerve into two separate fascicular bundles proximal to the carpal tunnel. The prevalence of BMN has been estimated to be approximately 10% in the general population and slightly higher among individuals with CTS [1].

Another anatomical variant that may contribute to carpal tunnel pathology is the persistent median artery (PMA). During embryonic development, the median artery serves as the principal vascular supply to the developing hand, but it typically regresses during the eighth week of gestation. Persistence of this vessel into adulthood may increase the volume of structures within the carpal tunnel and potentially contribute to median nerve compression [1].

BMN is frequently associated with a persistent median artery (PMA), and together these structures may increase the volume of tunnel contents and complicate both diagnostic interpretation and interventional planning. In CTS, recognition of BMN is important because cross-sectional area measurements must account for both nerve components, and failure to identify an accompanying PMA may increase procedural complexity during ultrasound-guided intervention. Accordingly, high-resolution ultrasound is particularly valuable in such cases because it allows direct visualization of the neural and vascular anatomy in real time. In the evaluation of CTS, high-resolution musculoskeletal ultrasound has become an increasingly valuable diagnostic modality because it allows direct visualization of nerve morphology, vascular structures, and dynamic nerve movement. Ultrasound findings in CTS should be interpreted within an integrated diagnostic framework that includes clinical history, physical examination, and electrodiagnostic testing when appropriate. Beyond morphologic assessment, ultrasound may also contribute through vascular and dynamic evaluation, particularly in cases with anatomical variants such as BMN and PMA. In addition, ultrasound enables precise guidance of interventional procedures and longitudinal assessment of structural changes. Conventional treatment strategies for CTS include conservative measures such as splinting and activity modification, corticosteroid injections, and surgical decompression. Although these treatments may provide symptomatic relief, they do not always address both the mechanical and inflammatory mechanisms contributing to nerve entrapment.

Ultrasound-guided hydrodissection has emerged as a minimally invasive technique that separates the nerve from surrounding tissues by injecting fluid into the perineural space, thereby releasing adhesions and restoring nerve mobility. Recent systematic reviews and randomized clinical trials have demonstrated promising outcomes using hydrodissection for CTS, suggesting that the technique may represent a viable alternative to surgery in selected patients [2,3].

Alpha-2 macroglobulin (A2M) is a large plasma glycoprotein that functions as a broad-spectrum protease inhibitor and cytokine scavenger. Through inhibition of proteolytic enzymes and modulation of inflammatory mediators, A2M has been proposed as a potential biologic adjunct for inflammatory and degenerative musculoskeletal conditions [1,4]. The present report describes an ultrasound-based diagnostic and interventional approach using hydrodissection with adjunctive A2M in a patient with symptomatic bifid median nerve entrapment associated with a persistent median artery.

## 2. Case Presentation

A 60-year-old right-handed woman presented with a one-year history of intermittent numbness, tingling, and pain involving the thumb, index finger, and middle finger of her dominant hand. The symptoms were exacerbated by repetitive hand activities and gripping tasks and were associated with nocturnal awakening due to paresthesia. The patient denied any prior wrist trauma, surgical intervention, or systemic conditions commonly associated with carpal tunnel syndrome.

Physical examination revealed decreased sensation in the thumb, index finger, and middle finger. Tinel’s sign over the carpal tunnel reproduced paresthesia radiating along the median nerve distribution, and Phalen’s maneuver similarly reproduced the patient’s symptoms. Grip strength measured using a standardized hand dynamometer was reduced to 12 kg. No thenar muscle atrophy or weakness of the abductor pollicis brevis was observed.

**Figure 1:**
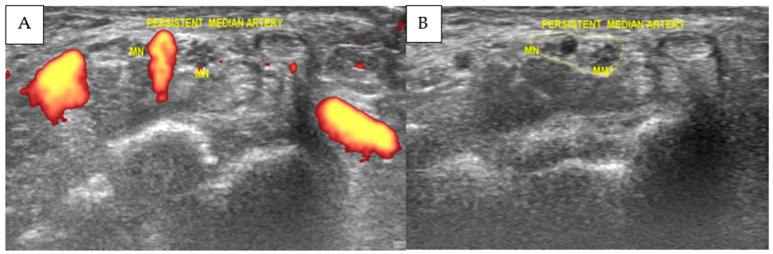
Transverse ultrasound image of the carpal tunnel showing a bifid median nerve (BMN) and a persistent median artery (PMA) with loss of the normal fascicular pattern (**A**). The combined cross-sectional area of the BMN was 0.19 cm^2^ (19 mm^2^), calculated as the sum of both nerve trunks (**B**). Power Doppler confirmed vascular flow within the PMA. MN, median nerve; PMA, persistent median artery.

High-resolution musculoskeletal ultrasound examination demonstrated a bifid median nerve accompanied by a persistent median artery and associated perineural edema within the proximal carpal tunnel, allowing detailed characterization of anatomical variants and nerve morphology. Power Doppler imaging confirmed vascular flow within the persistent median artery. The combined cross-sectional area of the bifid median nerve measured 0.19 cm^2^ (19 mm^2^), which exceeds commonly reported ultrasonographic thresholds used to support the diagnosis of CTS. In the setting of bifid median nerve anatomy, CSA was interpreted as the sum of both nerve trunks. These findings are illustrated in Figure 1, which demonstrates the transverse ultrasound appearance of the bifid median nerve and persistent median artery within the carpal tunnel. Based on the patient’s clinical presentation and ultrasound imaging findings, the diagnosis of symptomatic bifid median nerve entrapment associated with persistent median artery was established.

## 3. Materials and Methods

Following written informed consent, the patient underwent ultrasound-guided hydrodissection using autologous alpha-2 macroglobulin. Venous blood was collected and processed using the APEX filtration system/XCELL PC protein concentrator system (APEX Biologix, Clearwater, FL, USA), which isolates and concentrates the alpha-2 macroglobulin fraction from autologous plasma.

Ultrasound examination and the subsequent intervention were performed using a LOGIQ S8 ultrasound system equipped with an ML6-15 high-frequency linear transducer (6–15 MHz) (GE Ultrasound Korea, Ltd., Seongnam-si, Gyeonggi-do, Republic of Korea). The bifid median nerve and persistent median artery were identified at the carpal tunnel in both transverse and longitudinal planes, and Power Doppler imaging was used to confirm vascular flow within the persistent median artery. The cross-sectional area of the bifid median nerve was measured by tracing the inner border of the epineurium of each nerve trunk, and the combined cross-sectional area was calculated as the sum of both components. All procedures were performed under sterile conditions with continuous real-time ultrasound guidance by an operator with 18 years of experience in musculoskeletal ultrasound and ultrasound-guided interventions.

Local anesthesia was achieved with 2 mL of 1% lidocaine injected subcutaneously at the puncture site. A 23-gauge needle was advanced using an in-plane ultrasound technique toward the perineural space surrounding the bifid median nerve. Three milliliters of alpha-2 macroglobulin were then injected circumferentially around the nerve bundles. The injection created a fluid interface that separated the nerve from surrounding tissues and released perineural adhesions, which was continuously visualized under ultrasound.

The hydrodissection procedure was repeated once weekly for a total of three sessions.

## 4. Results

The patient tolerated all procedures without complications. Within one week of the first injection, the patient reported approximately a 20% reduction in pain intensity, although numbness persisted at that time. Three months after the final treatment session, the patient reported complete resolution of nocturnal paresthesia and substantial improvement in hand function during daily activities. 

**Figure 2:**
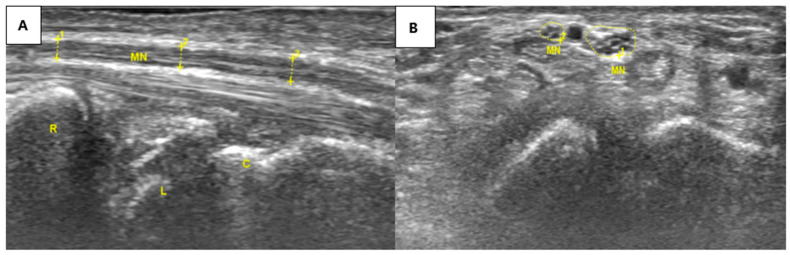
Ultrasound images obtained 10 months after the first treatment session. (**A**) Long-axis view showing a more uniform median nerve diameter. (**B**) Short-axis view showing restoration of a distinct fascicular pattern in the bifid median nerve. At this follow-up, the patient reported no numbness or pain in the affected wrist.

Follow-up ultrasound performed ten months after treatment demonstrated restoration of median nerve fascicular architecture and normalization of nerve morphology, indicating structural improvement on longitudinal imaging assessment. These findings are illustrated in Figure 2, which shows the normalized appearance of the median nerve in both short-axis and long-axis views. The patient reported no recurrence of symptoms during the follow-up period, with imaging findings consistent with sustained morphological improvement. 

**Figure 3:**
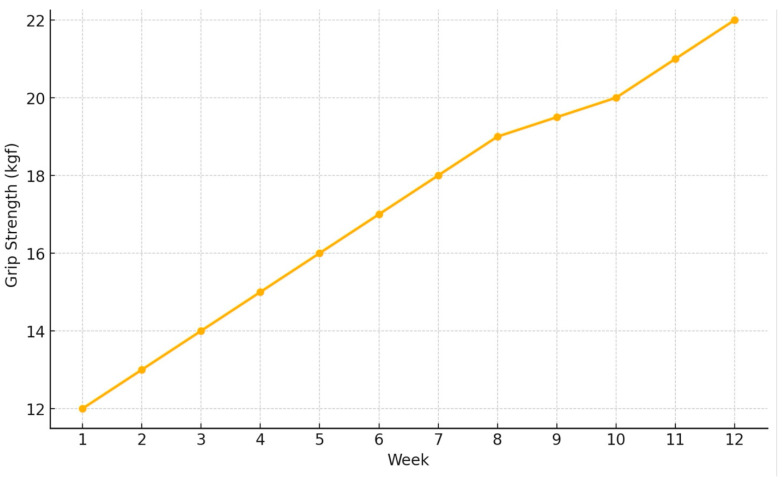
Serial grip strength measurements over the 12-week follow-up period.

Serial grip strength measurements (Figure 3 and Table 1) demonstrated progressive improvement over the treatment period. Baseline grip strength measured 12 kg. By week four, grip strength increased to 17 kg, and by week twelve, it reached 22 kg.

## 5. Discussion

This case report illustrates a potential clinical application of ultrasound-guided hydrodissection with autologous alpha-2 macroglobulin (A2M) in a patient with symptomatic carpal tunnel syndrome associated with the anatomical variants of a bifid median nerve (BMN) and a persistent median artery (PMA). The presence of these anatomical variations can complicate both the pathophysiology and treatment of carpal tunnel syndrome by increasing the volume of structures within the tunnel and altering the mechanical environment of the median nerve [5,6].

BMN is characterized by the division of the median nerve into two separate fascicular bundles proximal to the carpal tunnel. When combined with a persistent median artery, the effective cross-sectional area of the carpal tunnel contents may increase substantially, potentially contributing to nerve compression and impaired nerve gliding. Recognition of these variants is therefore essential during diagnostic imaging and interventional procedures [5,6].

In this case, ultrasound was essential for identifying the bifid median nerve and persistent median artery, guiding safe hydrodissection, and documenting interval morphologic improvement on follow-up imaging [7,8].

In recent years, ultrasound-guided hydrodissection has gained increasing attention as a minimally invasive therapeutic approach for peripheral nerve entrapment syndromes. The technique involves injecting fluid into the perineural space to mechanically separate the nerve from surrounding structures and release adhesions that restrict nerve mobility. By restoring the nerve’s natural gliding movement, hydrodissection may reduce mechanical irritation and potentially improve neural function [3,9].

Recent systematic reviews have reported encouraging outcomes with ultrasound-guided hydrodissection for CTS. A 2024 systematic review evaluating perineural hydrodissection for carpal tunnel syndrome reported that the technique can provide significant symptomatic relief while maintaining a favorable safety profile when compared with traditional injection therapies. The review concluded that hydrodissection represents a promising minimally invasive treatment option for CTS, although further randomized trials are needed to establish standardized protocols and determine optimal injectates [2,3].

More recent clinical research has added to the emerging evidence base for hydrodissection. A prospective randomized study comparing ultrasound-guided hydrodissection with open surgical release in patients with severe CTS demonstrated that hydrodissection can achieve meaningful symptom improvement while offering a less invasive treatment option for patients who wish to avoid surgery [10].

Additional randomized trials conducted in 2025 have explored variations in hydrodissection techniques and injectate composition. For example, a prospective double-blind randomized trial compared different injection volumes of 5% dextrose for ultrasound-guided hydrodissection in CTS and demonstrated significant improvements in functional outcomes and symptom severity scores across treatment groups [11].

Ongoing clinical trials are further investigating the comparative efficacy of various injectates, including corticosteroids and dextrose, delivered through ultrasound-guided hydrodissection. These studies highlight the growing interest in refining both the technique and the biologic components used in perineural hydrodissection for CTS [2,3].

Taken together, these findings suggest that hydrodissection may represent a reasonable minimally invasive option in selected patients with CTS, particularly in patients seeking a minimally invasive alternative to surgical decompression [2,3].

## 6. Biological Mechanisms of Alpha-2 Macroglobulin

While hydrodissection provides mechanical decompression of the median nerve, the addition of alpha-2 macroglobulin may act as a biologic adjunct with potential additional effects [1,12].

Alpha-2 macroglobulin is a large tetrameric plasma glycoprotein produced primarily by the liver and present in high concentrations in circulating plasma. It functions as a broad-spectrum protease inhibitor that neutralizes enzymes from multiple protease families, including serine, cysteine, and metalloprotease families [13,14].

The principal mechanism of A2M involves a unique “bait-and-trap” system. Proteases cleave a bait region within the A2M molecule, triggering a conformational change that physically traps the protease within a molecular cage. The resulting complex is subsequently recognized by macrophage receptors and cleared from circulation [13,14,15].

Beyond its role as a protease inhibitor, A2M has been shown to interact with cytokines, growth factors, and immune mediators. Through these interactions, A2M participates in the regulation of inflammation, tissue repair, coagulation pathways, and immune responses [14].

Several mechanisms may explain the potential therapeutic benefits of A2M in nerve entrapment syndromes. First, A2M inhibits matrix metalloproteinases (MMPs), enzymes that contribute to extracellular matrix degradation and fibrosis. Excessive MMP activity has been implicated in chronic inflammatory and degenerative musculoskeletal conditions [4,15]. Second, A2M can bind and neutralize pro-inflammatory cytokines such as tumor necrosis factor-alpha (TNF-α), interleukin-1 (IL-1), and interleukin-6 (IL-6). These cytokines are known to sensitize nociceptive neurons and contribute to the persistence of neuropathic pain [1,16,17]. Third, A2M functions as a carrier protein for multiple growth factors and signaling molecules, potentially facilitating tissue repair and regeneration within injured or inflamed tissues [14]. Finally, by modulating inflammatory signaling and reducing proteolytic activity, A2M may create a biological environment conducive to nerve recovery and restoration of normal neural function [1,12].

Although the patient improved clinically and sonographically after treatment, the present case does not allow separation of the mechanical effect of hydrodissection from any potential biologic effect of alpha-2 macroglobulin (A2M). Accordingly, A2M should be interpreted in this case as an exploratory adjunctive injectate and not as evidence of superiority or established efficacy relative to more commonly used agents such as dextrose or corticosteroids.

## 7. Clinical Implications and Comparison with Existing Therapies

The treatment of carpal tunnel syndrome typically progresses from conservative measures such as splinting and activity modification to corticosteroid injection and, ultimately, surgical decompression for persistent or severe cases [18,19].

Corticosteroid injections are widely used because they can provide rapid symptom relief. However, their therapeutic effect is often temporary, and repeated injections may carry risks such as tendon degeneration, tissue atrophy, and systemic corticosteroid exposure [20,21].

Surgical decompression remains the definitive treatment for severe CTS, particularly when thenar weakness or nerve conduction abnormalities are present [18,22]. Although surgical release has high success rates, it is associated with potential complications including scar formation, pillar pain, infection, and prolonged recovery [23,24].

Hydrodissection represents an intermediate treatment strategy that may offer several advantages. Because the procedure is minimally invasive, it can be performed in an outpatient setting with relatively low risk and short recovery time [3,9]. Additionally, ultrasound guidance improves procedural accuracy and reduces the risk of injury to adjacent structures [7,25]. In the present case, A2M was selected as an exploratory biologic adjunct based on its proposed protease-inhibitory and cytokine-modulating properties, while acknowledging that dextrose and corticosteroids remain more established injectates in current CTS hydrodissection practice.

Accordingly, biologic agents such as A2M may warrant further investigation as adjunctive injectates in hydrodissection, particularly with respect to inflammatory pathways relevant to nerve dysfunction [1,12].

As a single case report, the present findings are not generalizable and should be interpreted as preliminary. In addition, although grip strength was serially documented, additional objective outcome measures such as validated symptom questionnaires or electrodiagnostic follow-up were not available.

## 8. Conclusions

This case illustrates the potential value of ultrasound in the diagnosis, treatment guidance, and follow-up assessment of carpal tunnel syndrome in the setting of anatomical variants such as a bifid median nerve and persistent median artery. Ultrasound-guided hydrodissection using A2M was temporally associated with clinical and imaging improvement in this patient; however, the specific contribution of A2M cannot be determined from a single case report. These findings should therefore be considered preliminary and hypothesis-generating. Further controlled studies are needed to clarify the potential role of A2M as an adjunctive injectate in ultrasound-guided hydrodissection for CTS.

## Figures and Tables

**Table 1:** Serial grip strength measurements over 3 months (kgf).

Week	Grip Strength (kgf)
1	12.0
2	13.0
3	14.0
4	15.0
5	16.0
6	17.0
7	18.0
8	19.0
9	19.5
10	20.0
11	21.0
12	22.0

## Data Availability

The data presented in this study are available on reasonable request from the corresponding author.
